# Investigation of the risk factors in the development of radionecrosis in patients with brain metastases undergoing stereotactic radiotherapy

**DOI:** 10.1093/bjr/tqae051

**Published:** 2024-02-29

**Authors:** Bedriye Doğan, Harun Demir, Naciye Işık, Gun Gunalp, Hediye Pınar Günbey, Gökhan Yaprak

**Affiliations:** Department of Radiation Oncology, Faculty of Medicine, Inonu University, Malatya, Malatya, 44280, Turkey; Department of Radiation Oncology, Konya City Hospital, Konya, Konya, 42020, Turkey; Department of Radiation Oncology, Kartal Dr Lutfi Kırdar City Hospital, İstanbul, Istanbul, 34100, Turkey; Department of Radiation Oncology, Kartal Dr Lutfi Kırdar City Hospital, İstanbul, Istanbul, 34100, Turkey; Department of Radiology, Kartal Dr Lutfi Kirdar City Hospital, Istanbul, Istanbul, 34100, Turkey; Department of Radiation Oncology, Kartal Dr Lutfi Kırdar City Hospital, İstanbul, Istanbul, 34100, Turkey

**Keywords:** brain metastasis, radionecrosis, stereotactic radiosurgery, stereotactic radiotherapy

## Abstract

**Objective:**

To investigate the incidence, timing, and the factors predictors radionecrosis (RN) development in brain metastases (BMs) undergoing stereotactic radiotherapy (SRT).

**Methods:**

The study evaluated 245 BMs who exclusively received SRT between 2010 and 2020. RN was detected pathologically or radiologically.

**Results:**

The median of follow-up was 22.6 months. RN was detected in 18.4% of the metastatic lesions, and 3.3% symptomatic, 15.1% asymptomatic. The median time of RN was 22.8 months (2.5-39.5), and the rates at 6, 12, and 24 months were 16.8%, 41.4%, and 66%, respectively. Univariate analysis revealed that Graded Prognostic Assessment (*P* = .005), Score Index of Radiosurgery (*P* = .015), Recursive Partitioning Analysis (*P* = .011), the presence of primary cancer (*P* = .004), and localization (*P* = .048) significantly increased the incidence of RN. No significant relationship between RN and brain-gross tumour volume doses, planning target volume, fractionation, dose (*P* > .05). Multivariate analysis identified SIR > 6 (OR: 1.30, *P* = .021), primary of breast tumour (OR: 2.33, *P* = .031) and supratentorial localization (OR: 3.64, *P* = .025) as risk factors.

**Conclusions:**

SRT is used effectively in BMs. The incidence of RN following SRT is undeniably frequent. It was observed that the incidence rate increased as the follow-up period increased. We showed that brain-GTV doses are not predictive of RN development, unlike other publications. In study, a high SIR score and supratentorial localization were identified as factors that increased the risk of RN.

**Advances in knowledge:**

RN is still a common complication after SRT. Symptomatic RN is a significant cause of morbidity. The causes of RN are still not clearly identified. In many publications, brain dose and volumes have been found to be effective in RN. But, with this study, we found that brain dose volumes and fractionation did not increase the incidence of RN when brain doses were taken into account. The most important factor in the development of RN was found to be related to long survival after SRT.

## Introduction

Brain metastases (BMs) occur in ∼20%-40% of cancer patients over the course of their survival.^1^^,^^2^ The treatment options for BMs include surgery, chemotherapy, radiotherapy, and supportive therapies, used either individually or in combination, depending on the patient’s performance status and the histological characteristics of the primary tumour.^3^^,^^4^ Local recurrence is reported in 50%-60% of cases undergoing surgery alone,^5^^,^^6^ and so adjuvant whole brain radiotherapy (WBRT) or stereotactic radiotherapy (SRT) within the surgical cavity may be added to the treatment to reduce the risk.^6^^,^^7^ Quality of life and neurocognitive function may both decline as a result of WBRT.^6^ Randomized trials have reported SRT to be superior to WBRT in the preservation of neurocognitive function and to support the achievement of local control (LC).^7–9^ In general, SRT is the most common noninvasive treatment approach to metastatic lesions smaller than 3 cm and those without leptomeningeal dissemination, especially those located in areas not suitable for surgical intervention (such as the basal ganglion, brain stem, etc.).^3^^,^^4^

Stereotactic radiotherapy aims to achieve LC by delivering a high dose of radiation to the tumour while significantly reducing the radiation dose to the normal surrounding tissue.^8^^,^^9^ Various side effects may be observed after SRT, including radionecrosis (RN) as one of the most significant.^10–12^ Fischer and Holfelder (1930) referred to RN as parenchymal brain damage that affects vascular and glial cells at the radiotherapy site. The incidence is in the 3%-50% range,^13–17^ and diagnosis is based on histopathological and radiological imaging methods.^13–18^ RN is usually asymptomatic, while for symptomatic cases, the recommended treatments include surgery, bevacizumab therapy, corticosteroids (CSs), anticoagulant-antiaggregant therapies, hyperbaric oxygen therapy, and laser interstitial thermal therapy.^14^^,^^19–22^ Previous studies have reported RN to be influenced by various factors, including sex, the presence of comorbid conditions, performance status, tumour diameter and localization, prior brain therapies, chemotherapy, targeted therapies, conformity index (CI), homogeneity index (HI), dose and fractionation of stereotactic therapy, planning target volume (PTV), and the volumetric doses delivered to the brain.^11–13^^,^^15^^,^^23–26^

We present here the results of a retrospective examination of the incidence, timing, and risk factors associated with RN occurring after SRT in patients with BMs treated at our centre.

## Methods

The medical records of 455 patients who received SRT for BMs at Kartal Dr Lutfi Kirdar City Hospital between February 2010 and December 2020 were reviewed retrospectively. Included in the study were patients aged 18 years or older who had not undergone radiotherapy or surgery for BMs, and who had no extensive extracranial disease; while those with primary brain tumours or BMs associated with lymphoma, leukaemia, small cell lung cancer or germ cell tumours, and those with leptomeningeal involvement were excluded from the study. Also excluded from the analysis were patients who missed 2 consecutive MRI assessments following SRT. Consequently, the study was based on an analysis of the data of 245 BMs in 144 patients who met the above criteria who underwent SRT.

All patients were treated using a CyberKnife (CK) device. CK is a 6-arm robotic linear accelerator that performs stereotactic radiosurgery using 6 MV X-rays. It has 2 important advantages over other radiosurgery devices. First; No invasive stereotactic fixation frame is used to the patient’s skull for fixation, just a simple thermoplastic mask. The second is that it contains a nonisocentric linac system.

Patient treatment planning: The gross tumour volume (GTV) was determined from contour measurements of the tumour identified on contrast-enhanced T1A MRI sequences of the patients, and the PTV was established based on a 1-mm margin around the GTV. The following doses that are commonly used at our clinic were assigned to the PTV: 20-22 Gy/1 fraction (fx) for lesions <1 cm, 24 Gy/2fx for 1-2 cm, 27 Gy/3fx for 2.1-3 cm, 28-32 Gy/4fx for 3.1-4 cm, and 30-35 Gy/5fx for 4.1-5 cm. The treatment plans were established using the MultiPlan (Accuray, Inc.) planning system, as per ICRU 91 standards.^27–29^ The normal brain tissue doses evaluated in the present study (8, 10, 12, 14, 16, 18, 21 Gy) were determined by subtracting the GTV from the whole brain doses. All patients had conventional MRIs with 1 mm section, T1 contrast, T2 and diffusion sequences taken for pretreatment SRT planning and posttreatment evaluation and follow-up (at 3-month intervals).

Response to therapy was assessed from MRI scans as per the Response Assessment Neuro-Oncology criteria, while toxicity was assessed using the Radiation Therapy Oncology Group central nervous system toxicity scoring system.^30^^,^^31^

**Figure 1.**
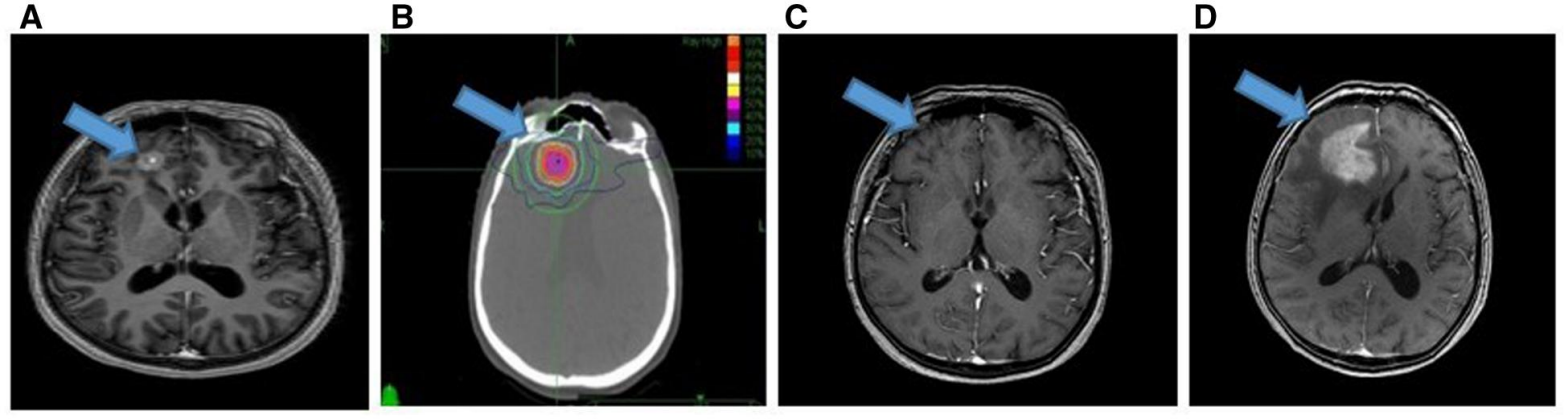
(A) T1W brain MRI sequence shows heterogeneous contrast enhancement in the right frontal region, consistent with a 12-mm diameter metastasis. (B) CT image of SRT planning. (C) MRI T1A sequence image 6 months after treatment; complete response. (D) MRI image at the 12th month after treatment. Pathologically confirmed MRI T1A sequence of radionecrosis image with peripheral oedema in the right frontal region, contrast enhancement in the middle and soap bubble appearance.

The diagnosis of RN was based on the pathological examination or imaging findings. RN was determined radiologically by a single neuroradiologist with long experience in the field. Radiological RN imaging findings: (1) Increased oedema at the radiotherapy site in T2-weighted contrast-enhanced conventional MRI sequences and increased contrast uptake in T1A-weighted sequences (Figure 1), (2) stable lesions or spontaneous regression and lack of progression in consecutive follow-up imaging studies (3-6 months), (3) diffusion restriction in Magnetic Resonance-Diffusion Weighted Imaging and Apparent Diffusion Coefficient mapping, (4) decreased perfusion at the centre of the lesion with contrast enhancement in MR perfusion, and (5) decreases in N-acetyl aspartate, choline, and creatinine values and peaks in lipid and lactate values on MR-Spectron.^23^^,^^32^

The data were analysed using IBM SPSS Statistics (Version 22.0. Armonk, NY: IBM Corp.). A logistic regression analysis was used to analyse the relationships between variables, a Kaplan-Meier test was used for the survival analysis, and a *P*-value of <.05 was considered significant.

The study received approval from the Istanbul Kartal Dr Lütfi Kırdar City Hospital Clinical Trials Ethics Committee with protocol number 514/192/18, dated December 30, 2020.

## Results

### Patient, metastasis, and treatment characteristics

Included in the study were 144 patients who met the study criteria in whom 245 metastases were examined. The patient, metastasis, and treatment characteristics at the time of SRT are presented in Table 1. Of the study patients, 38.2% (*n* = 55) were female and 61.8% (*n* = 89) were male, and the median age was 59 years (29-90 years). The primary tumour was nonsmall cell lung cancer (NSCLC) in 56.9% of the patients, breast cancer in 20.1%, and genitourinary cancer in 10.5%. The Recursive Partitioning Analysis (RPA) prognostic score was I in 13.9% (*n* = 20) and II in 86.1% (*n* = 124), while no patient had an RPA score of III. The median Graded Prognostic Assessment (GPA) score was 2 (0.5-3.5) and the median Score Index of Radiosurgery (SIR) was 6 (2-10). Of the treated 245 metastases, 29.8% (*n* = 73) were localized in the parietal lobe, 20.8% (*n* = 51) in the cerebellum, 18.8% (*n* = 46) in the frontal lobe, 16.3% (*n* = 40) in the occipital lobe, and 14.3% (*n* = 35) in the temporal lobe, while 80% (196) of the lesions had supratentorial and 20% (*n* = 49) had infratentorial localizations. SRT was administered at a median dose of 20 Gy (15-25.5), and of the metastases, 62.4% (*n* = 153) received 1 fraction, 32.2% (*n* = 79) received 2 fractions, 4.9% (*n* = 12) received 3 fractions, and 0.4% (*n* = 1) received 5 fractions. The median PTV of the metastases was 4742.18 mm^3^ (11.22-45 055), while the median whole brain dose was 134.71 cGy (18.44-7014). The median brain-GTV dose volumes that received doses of 8, 10, 12, 14, 16, 18, and 21 Gy were (V8-21) 23.08 cm^3^ (1.34-234.71), 16.31 cm^3^ (0.97-182.9), 12.58 cm^3^ (0.73-153.43), 9.80 cm^3^ (0-134.09), 7.44 cm^3^ (0-114.93), 5.26 cm^3^ (0-95.74), and 2.27 cm^3^ (0-93), respectively.

**Table 1.** Patient and metastasis characteristics.

Variables	Value	*N* %
**Patient characteristics (*n* = 144)**		
**Age**		
Median (min-max)	59 (29-90)	
**Gender**		
Female	55	38.2
Male	89	61.8
**The origin of primary disease**		
NSCLC	82	56.9
Breast	29	20.1
Genitourinary tract	15	10.5
Other	18	12.6
*Performance scores*		
**RPA**		
I	20	13.9
II	124	86.1
**GPA** median (min-max)	2 (0.5-3.5)	
**SIR** median (min-max)	6 (2-10)	
**Metastasis characteristics (*n* = 245)**		
**Metastasis diameter (mm)**		
Mean ± SD	13.58 ± 9.15	
(min-max)	(3-50)	
**Brain lobe localization**		
Parietal	73	29.8
Cerebellar	51	20.8
Frontal	46	18.8
Occipital	40	16.3
Temporal	35	14.3
**Tentorial localization**		
Supratentorial	196	80.0
Infratentorial	49	20.0
**Treatment characteristics (*n* = 245)**		
**SRT PTV (mm^3^)**	4742.18 ± 6983.05 (11.22-45,055)	
**SRT dose (Gy)**	20 (15-25.5)	
**CI**	1.27 ± 0.95 (1-13.78)	
**BED 3**	126.65 ± 30.22 (66.67-183.33)	
**Whole brain mean dose (cGy)**	134.71 ± 447.90 (18.44-7014)	
**Brain-GTV dose volumes (cm^3^)**		
**V8**	23.08±31.55 (1.34-234.71)	
**V10**	16.31±21.50 (0.97-182.9)	
**V12**	12.58±17.23 (0.73-153.43)	
**V14**	9.80±14.18 (0-134.09)	
**V16**	7.44±11.56 (0-114.93)	
**V18**	5.26±9.13 (0-95.74)	
**V21**	2.27±7.94 (0-93)	

Abbreviations: BED=biologically effective dose, CI=conformity index, DM=diabetes mellitus, GPA=graded prognostic assessment, HI=homogeneity index, NSCLC=nonsmall cell lung cancer, nCI=new conformity index, RPA=recursive partitioning analysis, SIR=score index of radiosurgery, SRT=stereotactic radiotherapy, PTV=planning target volume.

### LC and RN characteristics

The median duration of follow-up following SRT was 22.6 months (3.7-116.9), and the median survival time was 33.8 months. The median duration of LC was 26 months, while the rate of LC at 1 year was 71.1%. Of patients with radiological progression, 47.1 (*n* = 40) were reported as pure tumour progression, 32.9% (*n* = 28) as only RN, and 20% (*n* = 17) as both necrosis and progression. Of the 15 lesions operated on due to radiological progression or the development of symptoms, tumour progression was detected in 66.7% (*n* = 19) and RN was detected in 33.3% (*n* = 5). RN occurred in 23.6% (*n* = 34) of the patients and 18.4% (*n* = 45) of metastases, while 3.3% (*n* = 8) were symptomatic and 15.1% (*n* = 37) were asymptomatic. The median time to the development of RN was 22.8 months (2.5-39.5 months), and the rates of RN at 6, 12, 18, and 24 months were 16.8%, 41.4%, 52.9%, and 66%, respectively. RN occurred in 18.3% of the 153 metastases in the single fraction group and in 18.5% of the 92 metastases in the multiple fraction (2, 3, and 5 fx) group. Of the lesions with asymptomatic RN, 80% (*n* = 36) were observed without intervention. For symptomatic lesions or those showing progression despite being asymptomatic, 11.1% (*n* = 5) underwent surgery and 8.9% (*n* = 4) received CS therapy.

The study identified no significant relationship between RN and gender, primary tumour histology, metastasis localization according to brain layer, fractionation, stereotactic dose, PTV, biologically effective dose (BED) 3-10, brain mean dose, and brain-GTV doses (*P* > .05).

In a univariate analysis, a GPA index >2 (*P* = .005), an SIR >6 (*P* = .015), an RPA score of I (*P* = .011), the presence of primary breast cancer (*P* = .004), and a supratentorial localization (*P* = .048) significantly increased the incidence of RN, while factors such as occipital lobe localization (*P* = .051) and patient’s age below 60 years (*P* = .05) showed borderline statistical significance for the development of RN (Table 2). Furthermore, a significant relationship was found between V21 and RN in the asymptomatic RN group (*P* = .02), and a V21 >1.5 cm^3^ increased the risk of developing RN.

**Table 2.** Univariate and multivariate analysis of factors predicting radionecrosis.

	Univariate analysis		Multivariate analysis	
	OR (95% CI)	*P*-value	OR (95% CI)	*P*-value
**Age**	0.972 (0.944-1.001)	.058		
**The origin of primary disease**		**.004**		**.003**
Breast/NSCLC	2.126 (1.021-4.427)	**.044**	2.337 (1.081-5.051)	**.031**
Other/NSCLC	0.248 (0.072-0.857)	**.027**	0.250 (0.071-0.874)	**.030**
**SIR**	1.308 (1.053-1.625)	**.015**	1.301 (1.040-1.627)	**.021**
**GPA**	2.015 (1.236-3.286)	**.005**		
**RPA (I/II)**	2.798 (1.269-6.171)	**.011**		
**Brain lobe localization**		.051		
Occipital/frontal	1.532 (0.594-3.949)	.377		
Parietal/frontal	0.626 (0.250-1.567)	.317		
Temporal/frontal	0.411 (0.119-1.422)	.160		
Cerebellum/frontal	0.346 (0.110-1.087)	.069		
**Tentorial localization** (supratentorial/infratentorial)	2.976 (1.012-8.754)	**.048**	3.644 (1.176-11.288)	**.025**

Abbreviations: NSCLC = nonsmall cell lung cancer, GPA = graded prognostic assessment, SIR = score index of radiosurgery, RPA = recursive partitioning analysis. Values less than p:0.05 were considered significant. Significant parameters are written in bold.

In a multivariate analysis, SIR (OR: 1.30, 95% CI, 1.040-1.627, *P* = .021), the presence of primary breast cancer (OR: 2.33, 95% CI, 1.08-5.05, *P* = .031) and a supratentorial localization (OR: 3.64, 95% CI, 1.176-11.28, *P* = .025) were all identified as risk factors related to RN.

## Discussion

Efforts to reduce the side effects of radiotherapy have recently gained importance owing to enhanced survival rates resulting from new chemotherapeutics, immunotherapy, targeted therapies, and improved brain control via stereotactic therapy.^3^^,^^4^ The underlying mechanisms of RN—a significant side effect following SRT—have yet to be fully elucidated, although its pathophysiology has been attributed to vascular and glial cells, immunological mechanisms, and free radical damage.^14^^,^^16^^,^^17^ Previous studies analysing brain RN reporting the incidence rates have suggested numerous approaches to the prediction of RN development.^13^^,^^23^^,^^33^^,^^34^

In this retrospective study, the incidence of RN was 18.4%, of which 3.3% was symptomatic and 15.1% was asymptomatic, while previous studies in the literature have reported incidence rates of 3%-50% for RN following SRT and 2%-14% for symptomatic RN.^15^^,^^16^^,^^25^^,^^33^ While the incidence rate for RN in the present study is consistent with those reported in the literature, the rate of symptomatic RN is lower than those reported in previous studies. Of the patients in our study group, 30.6% (*n* = 44) received CS therapy during SRT, which is thought to reduce both the overall the incidence of RN by suppressing immunological events, and the incidence of symptomatic RN due to its antioedema effects. In an earlier study in 1959, Lampert^17^ hypothesized that pathological changes occurring in RN may be attributable to an antibody response to the antigens released from damaged glial cells, while Ryken et al^35^ reported that CS therapy reduced the acute or subacute effects of radiotherapy.

In the present study, the median time to the development of RN was 22.8 months (2.5-39.5 months), while the incidences of RN at 6, 12, 18, and 24 months were 16.8%, 41.4%, 52.9%, and 66%, respectively. We found that the cumulative incidence of RN increased with the duration of follow-up, and also that a GPA index >2, an SIR >6, an RPA of I, the presence of primary breast cancer, and age below 60 years significantly increased the incidence of RN. We believe that the expected survival in these patients is longer, which allows sufficient time for the development of RN, contributing to the greater incidence of RN. While there is a scarcity of data in the literature, Minniti et al^15^ identified KPS and RPA as prognostic factors for the development of RN, while Leyrat et al^34^ reported no prognostic value for SIR, RPA, GPA, or DS-GPA. Sneed et al^13^ reported a higher incidence of RN among patients with primary kidney tumours, whereas Kohutek et al^36^ detected RN at a higher rate in primary cancers other than NSCLC, melanoma, and breast cancer.

In the present study, the supratentorial localization of the tumour was identified as a factor that increased the risk of RN, while occipital lobe localization showed borderline statistical significance. Previous studies in the literature have reported a relationship between tumour localization and the development of RN, although there is conflicting data on which tumour localization exhibits the strongest relationship with RN. Minniti et al^15^ reported that a parietal lobe localization predicted the development of RN, in contrast to Kortyko et al’s^25^ suggested occipital and temporal lobe localization.

In the present study, the brain doses reported to be influential in the development of RN in previous studies were not identified as predictive factors, as in the asymptomatic RN subgroup only V21 was found to be associated with the development of RN. In our clinic, particular emphasis is placed on keeping doses below specific thresholds, such as 12, 16.2, 19.6, 21.5, and 24.4 Gy, for brain-GTV volumes of 10 cm^3^ at 1, 2, 3, 4, and 5 fractions, respectively, in patients undergoing SRT for BMs. Considering the careful attention we devote to these limitations, we believe that dosimetric risk factors have no predictive value for the development of RN. In a retrospective study conducted by Leyrat et al^34^ in 2022 involving patients with BMs associated with NSCLC, no significant relationship was reported between brain-GTV doses and volumes and the development of RN, similar to our study. Blonigen et al^11^ reported V8, 10, 12, and 16 as risk factors determining the development of symptomatic RN, with a cut-off value of 10.45 cm^3^ for V10 and 7.85 cm^3^ for V12, whereas Minniti et al^15^ reported a V10 >12.6 cm^3^ and a V12 >10.9 cm^3^ to be associated with a significantly increased risk of RN. Even though the mean brain-GTV volumes in our patient group were similar to those reported in the aforementioned studies, no such association with the development of RN was identified. Furthermore, in the present study, brain doses were examined using volumetric data after excluding the GTV values from the brain tissue, while the doses and volumes studied in the literature are variable. Predicting the development of RN based on dose-volume parameters is even more challenging, primarily because previous studies have reported dose-volume values associated with increased risk based on various cut-off values.^11^^,^^13^^,^^15^^,^^25^^,^^26^^,^^34^ The studies reporting the development of RN following SRT and the factors predicting the development of RN are presented in Table 3.

**Table 3.** Stereotactic radiotherapy and radionecrosis studies.

Study	Device	Number of patients/metastases	Dose/Fx	Previous therapy	Time to the development of RN	RN risk symptomatic asymptomatic	Related factors	Brain volume development
Blonigen et al^11^	LINAC-based SRS	63/173 Brain metastasis	Mean 18/1 HI: 1.22 CI: 2.45	63% WBRT	**—**	14% 10% S-RN 4% AS-RN	CIV8-16 **Symp. RN;** **V8-16** V10 > 10.45 cm^3^ V12 > 7.85 cm^3^	Not mentioned
Sneed et al^13^	Gamma Knife	435/2200 Brain metastasis	**—**	WBRT/SRS	**—**	5.4%	WBRT/SRS Tumour volume V12 Gy RCC	Whole brain
Minniti et al^15^	LINAC-based SRS	206/310 Brain metastasis	Mean18/1 HI: 1.1 CI: 1.6	NA	11-10 months	24% 10% S-RN 14% AS-RN	KPS, RPA Parietal lobe Tumour volume V10-16 **S-RN;** V10 > 12.6 cm^3^ V12 > 10.9 cm^3^	Not mentioned
Kortyko et al^25^	Gamma Knife	129/198 Brain metastasis/ primary tumour	Mean 17/1	WBRT	**—**	**—**	**S-RN**; Occipital lobe Temporal lobe Male gender WBRT **V12 > 10 cm^3^**	Whole brain
Sayan et al^26^	LINAC-based SRS/SRT	170/323 Brain metastasis	**—**	**—**	—	7% S-RN	**S-RN;** Single fx, DM, PTV, V50%, V10%, V10, V12	Whole brain
Leyrat et al^34^	LINAC-based HSRT	87/101 NSCLC Brain metastasis	Mean 23.1/3	WBRT	**—**	5.9% S-RN	Dyslipidemia **V8-21 not related	Brain-GTV
Kohutek et al^36^	LINAC-based SRS	160/271 Brain metastasis	**—**	WBRT	**—**	25.8% 17.3% S-RN 8.5% AS-RN	Tumour diameter History of WBRT, prescription, NSCLC, breast, nonmelanoma	Not mentioned

Abbreviations: S-RN = symptomatic radionecrosis, AS-RN = asymptomatic radionecrosis, Fx = fraction, WBRT = whole brain radiotherapy, PTV = planning target volume.

** Leyrat et al reported in their study that dose-volumes up to V8-21 were not associated with RN.

Literature also contains studies reporting a relationship between RN and the target volume, fractionation, tumour diameter, and CI.^11^^,^^13^^,^^15^^,^^26^^,^^36^ which were not identified as predictive factors in the present study. The mean radiotherapy dose and BED3 value, which is crucial for the assessment of late-term toxicity, were 19.5 and 147.4 Gy in the single-fraction group, and 21.6 and 92 Gy in the multifraction group. The present study identified no relationship between the dose and fractionation and the development of RN. In a study conducted by Loo et al reporting mean BED3 and radiotherapy doses of 79.3 and 14 Gy in the single-fraction group, and doses of 82.4 and 23.31 Gy in the multifraction group, the incidence of RN was found to be higher in the single-fraction group than in the multifraction SRT group (1% vs 6.4%). The authors attributed this finding to the inclusion of large tumours in the multifraction group and SRT dosed as low as 14 Gy in the single-fraction group.^33^ In their study, Donovan et al^12^ reported the incidence rates for RN in the single- and multifraction SRT groups (1 vs 3 fractions, OR: 1.0, 95% CI, 0.3-3.6, *P*=.98) that were consistent with the data of the present study. In a study reporting CI as a predictive factor for the development of RN, Blonigen et al^11^ reported a mean CI of 1.9, while Minniti et al^15^ reported a mean CI of 1.6. Paddick defined CI—a crucial parameter in the planning of stereotactic therapy—as the ratio of the squared target volume covered by the prescribed dose both to the treatment volume and the prescribed isodose volume. If the CI falls within the 1-2 range, even though it may align with the treatment plan, the goal is to bring the CI value as close to 1 as possible in the planning of SRT.^27^^,^^29^ A CI value close to 1 indicates that the delivered dose perfectly covers the target volume while minimizing the dose delivered to healthy brain parenchyma. A sharp decrease in the dose delivered to the normal tissue reduces the likelihood of brain damage caused by RT.^11^^,^^15^^,^^27^ The mean CI in the present study was 1.27, and no relationship with the development of RN was identified.

In conclusion, although the incidence of RN is high, it is often not adequately reflected in clinical practice. RN is more frequently diagnosed due to the increased accessibility to radiological imaging methods and the longer follow-up durations associated with improved survival in patients with primary breast cancer and high RPA-I, GPA, and SIR.

There is a need for multicentre, prospective studies to investigate the pathophysiology of RN and to identify the predictive risk factors.

## Data Availability

Data sets were analysed.
